# A Novel Soybean Dirigent Gene *GmDIR22* Contributes to Promotion of Lignan Biosynthesis and Enhances Resistance to *Phytophthora sojae*

**DOI:** 10.3389/fpls.2017.01185

**Published:** 2017-07-04

**Authors:** Ninghui Li, Ming Zhao, Tengfei Liu, Lidong Dong, Qun Cheng, Junjiang Wu, Le Wang, Xi Chen, Chuanzhong Zhang, Wencheng Lu, Pengfei Xu, Shuzhen Zhang

**Affiliations:** ^1^Key Laboratory of Soybean Biology of Chinese Education Ministry, Soybean Research Institute, Northeast Agricultural UniversityHarbin, China; ^2^Jiamusi Branch of Heilongjiang Academy of Agricultural SciencesJiamusi, China; ^3^Key Laboratory of Soybean Cultivation of Ministry of Agriculture China, Soybean Research Institute, Heilongjiang Academy of Agricultural SciencesHarbin, China; ^4^Heihe Branch of Heilongjiang Academy of Agricultural SciencesHeihe, China

**Keywords:** *Glycine max*, dirigent protein, lignan, *Phytophthora sojae*, gene expression

## Abstract

Phytophthora root and stem rot caused by the oomycete pathogen *Phytophthora sojae* is a destructive disease of soybean worldwide. Plant dirigent proteins (DIR) are proposed to have roles in biosynthesis of either lignan or lignin-like molecules, and are important for defense responses, secondary metabolism, and pathogen resistance. In the present work, a novel *DIR* gene expressed sequence tag is identified as up-regulated in the highly resistant soybean cultivar ‘Suinong 10’ inoculated with *P. sojae*. The full length cDNA is isolated using rapid amplification of cDNA ends, and designated *GmDIR22* (GenBank accession no. HQ_993047). The full length *GmDIR22* is 789 bp and contains a 567 bp open reading frame encoding a polypeptide of 188 amino acids. The sequence analysis indicated that GmDIR22 contains a conserved dirigent domain at amino acid residues 43–187. The quantitative real-time reverse transcription PCR demonstrated that soybean *GmDIR22* mRNA is expressed most highly in stems, followed by roots and leaves. The treatments with stresses demonstrated that *GmDIR22* is significantly induced by *P. sojae* and gibberellic acid (GA_3_), and also responds to salicylic acid, methyl jasmonic acid, and abscisic acid. The GmDIR22 is targeted to the cytomembrane when transiently expressed in Arabidopsis protoplasts. Moreover, The GmDIR22 recombinant protein purified from *Escherichia coli* could effectively direct *E*-coniferyl alcohol coupling into lignan (+)-pinoresinol. Accordingly, the overexpression of *GmDIR22* in transgenic soybean increased total lignan accumulation. Moreover, the lignan extracts from *GmDIR22* transgenic plants effectively inhibits *P. sojae* hyphal growth. Furthermore, the transgenic overexpression of *GmDIR22* in the susceptible soybean cultivar ‘Dongnong 50’ enhances its resistance to *P. sojae*. Collectively, these data suggested that the primary role of GmDIR22 is probably involved in the regulation of lignan biosynthesis, and which contributes to resistance to *P. sojae*.

## Introduction

Dirigent proteins (DIR), whose name is derived from the Latin word, *dirigere* (to align or guide), were first identified in *Forsythia suspensa* (Davin et al., 1997). The dirigent gene family has several members in numerous plant species, including lichens, ferns, gymnosperms, and angiosperms (Ralph et al., 2006, 2007; Wu et al., 2009). Many dirigent genes are inducible by different types of abiotic and biotic stress factors, including wounding (Ralph et al., 2007), dehydration, low temperature, abscisic acid (ABA) (Wu et al., 2009), H_2_O_2_, NaCl, and polyethylene glycol (Magalhaes et al., 2010). The apparent upregulation of dirigent genes in response to attacks by fungi (Zhu et al., 2007; Reboledo et al., 2015) and insects (Ralph et al., 2007) are of particular interest. The predominant roles of the dirigent gene family are in defense responses, secondary metabolism, and fiber biosynthesis (Davin and Lewis, 2003; Ralph et al., 2007; Berim et al., 2008). Moreover, they have important roles in plant secondary metabolism, including contribution to lignan and lignin formation (Liu et al., 2008; Pickel et al., 2010; Kim H.J. et al., 2012; Dalisay et al., 2015; Effenberger et al., 2015). In addition, DIR have been implicated in certain developmentally controlled lignification processes, where they contribute to the formation of the casparian strip in the developing Arabidopsis root (Hosmani et al., 2013).

Groundbreaking *in vitro* biochemical analysis by Davin et al. (1997) demonstrated that a *Forsythia intermedia* dirigent protein (FiDIR) could stereoselectively couple two coniferyl alcohol molecules to produce a (+)-pinoresinol in the presence of an oxidase or electron oxidant. The same specificity has been reported for DIRs from *Thuja plicata* (TpDIR5, 8) (Kim et al., 2002), *Schisandra chinensis* (ScDIR) (Kim K.W. et al., 2012), *Pisum sativum* (PsDRR206) (Seneviratne et al., 2015), and *Linum usitatissimum* (LuDIR1) (Dalisay et al., 2015). Coniferyl alcohol is the direct precursor for formation of lignan dimers. Meanwhile, coniferyl and sinapyl alcohol, together with other monolignol *p*-coumaryl alcohols, are also polymerized into the macromolecule lignin, certain biosynthetic pathways overlap in production of lignans and lignin (Davin and Lewis, 2003). These two major classes of plant metabolic products are apparently unique to vascular land plants, and together they constitute approximately 20–30% of their organic carbon (Lewis and Yamamoto, 1990; Lewis and Davin, 1998, 1999; Lewis et al., 1999). Lignans are structurally defined as 8, 8′-coupled phenylpropanoid dimers (Halls et al., 2004; Davin and Lewis, 2005), their primary formation can be under either constitutive or inducible control, and their composition and amounts vary markedly among plant species (Lewis and Davin, 1999). Lignans are present in different tissues and organs of vascular plants (roots, stems, leaves, flowers, seeds, etc.) (Ayres and Loike, 1990), and have potent plant defense properties, including antiviral activities (Chang et al., 1995), antioxidant (Lundberg et al., 1944; Osawa et al., 1985), antifeedant (Matsui and Munakata, 1975), and antimicrobial (Nakatani et al., 1988; Pauletti et al., 2000; Harmatha and Dinan, 2003; Saleem et al., 2005), which are increasingly considered of practical importance (MacRae and Towers, 1984; Lewis and Davin, 1999). Lignin is the main component of plant cell walls, and has an important role in development. Accumulation of lignin and callose in the cell walls enhances plants mechanical strength and prevents pathogen spread between cells (Tiburzy and Reisener, 1990). The lignin formed in response to pathogens or elicitors is significantly different from the type that occurs in undamaged plant structures; therefore, it has been speculated that the regulation of lignin synthesis in defense responses and development may be controlled by different pathways (Lange et al., 1995; Pomar et al., 2002).

The dirigent phenomenon was discovered by Davin et al. (1997), who studied the coupling of two coniferyl alcohol radicals generated from coniferyl alcohol by single-electron transfer (e.g., by laccases or peroxidases) as the first step in the biosynthesis of lignans (Davin and Lewis, 2000). Although DIR were discovered more than a decade ago, the biological functions of many DIR proteins remain ambiguous, and direct experimental evidence substantiating the contributions of DIR to lignin biosynthesis is rare (Önnerud et al., 2002; Davin et al., 2008; Ma and Liu, 2015).

Phytophthora root and stem rot of soybean (PRR), caused by the oomycete pathogen *Phytophthora sojae*, is a destructive soil-borne disease of soybean all around the world (Wrather et al., 1997; Tyler, 2007). In our previous study, a cDNA library was constructed from the leaf tissues of the highly resistant soybean ‘Suinong 10,’ using suppression subtractive hybridization, and the mRNAs encoding expressed sequence tags (ESTs) showed increased expression during *P. sojae* infection (Xu et al., 2012). In this study, a full-length cDNA corresponding to an up-regulated EST encoding a conservative dirigent domain was isolated from soybean ‘Suinong 10’ using rapid amplification of cDNA ends (RACE). The full-length cDNA belonged to a new member of the soybean DIR family and was designated *GmDIR22* (GenBank accession no. HQ_993047). The expression patterns of the *GmDIR22* gene were characterized in response to abiotic and biotic stresses, and its expression levels were determined in different soybean tissues. GmDIR22 protein expressed in *Escherichia coli* was purified, and its function was investigated. Furthermore, the content of lignan and lignin in transgenic plants overexpressing *GmDIR22* was established and the antimicrobial activity of lignan extracts against *P. sojae* hyphal growth was evaluated. Moreover, the transgenic soybean plants over-expressing *GmDIR22* gene were produced, and their antimicrobial properties were also investigated.

## Materials and Methods

### Plant Materials and Growth Conditions

The resistant soybean cultivar ‘Suinong 10’ against the predominant race of *P. sojae* (race 1) in Heilongjiang, China (Zhang et al., 2010), was used for gene isolation and the seedlings at the first-node stage (V1; Fehr et al., 1971) were used for various treatments. *P. sojae* race 1, PSR01, isolated from infected soybean plants in Heilongjiang (Zhang et al., 2010), was used in the experiment. ‘Suinong 10’ seeds were grown with a photoperiod of 16/8 h light/dark and maintained at 22°C and 70% relative humidity in a greenhouse. The susceptible soybean cultivar ‘Dongnong 50’ was obtained from Key Laboratory of Soybean Biology of Chinese Education Ministry, Harbin, and used for gene transformation experiments.

### Isolation and Sequence Analysis of *GmDIR22*

Rapid amplification of cDNA ends was performed to isolate the full-length cDNA, corresponding to an EST up-regulated in soybean ‘Suinong 10’ in response to *P. sojae* infection, using the SMART RACE cDNA amplification Kit (Clontech, CA, United States). The gene-specific primers GSP1 for 5′- RACE and GSP2 for 3′-RACE (and) were designed to produce the antisense and sense strands, respectively. The full-length cDNA sequence of *GmDIR22* was obtained by PCR amplification from ‘Suinong 10’ cDNA using the primer pair *GmDIR22F* and *GmDIR22R* (Supplementary Table S1). PCR products were ligated into the pMDTM18-T vector (Takara Biotech Inc, Dalian, China), then transformed into *E. coli* DH5α cells (Shanghai Biotech Inc, Shanghai, China) and sequenced (GENEWIZ, Beijing, China).

NCBI bioinformatics tools were used to analyze nucleotide and protein sequence data^1^. The predicted protein structure was analyzed using SMART^2^. Sequence alignments were performed using DNAMAN software^3^. A phylogenetic tree, based on the nucleotide sequences of GmDIR22 and other DIR members, was generated using MEGA 5.1 software^4^. The three-dimensional structure of GmDIR22 was predicted using the online program, Phyre2^5^.

### Stress Treatments

To investigate *GmDIR22* gene expression patterns in response to various stress factors, ‘Suinong 10’ soybean seedlings were treated with 50 mg.L^-1^ gibberellic acid (GA_3_), 100 μM methyl jasmonic acid (MeJA), 0.5 mM salicylic acid (SA), and 50 mM ABA. The unifoliolate leaves were harvested at 0, 3, 6, 9, 12, and 24 h after the treatment, wrapped in aluminum foil, immediately frozen in liquid nitrogen, and stored at –80°C until RNA extraction and cDNA synthesis. The untreated soybean leaves were used as controls. For *P. sojae* treatment, soybean plants were infected with *P. sojae* zoospores following the method described by Ward et al. (1979). The unifoliolate leaves were harvested for RNA isolation at 0, 6, 12, 24, 48, and 72 h after the treatments, wrapped in aluminum foil, immediately frozen in liquid nitrogen and stored at –80°C.

### Quantitative RT-PCR Analysis

To determine the abundance of the *GmDIR22* transcript, qRT-PCR analysis was performed. Total RNA was extracted from ‘Suinong 10’ soybean leaves at different time points after diverse stress treatments using TRIzol reagent (Invitrogen, Shanghai, China). First-strand cDNA was synthesized from 1 μg RNA using a Moloney murine leukemia virus reverse transcriptase kit (Takara, Dalian, China), according to the manufacturer’s protocol. qRT-PCR analysis was performed using a real-time RT-PCR kit (Takara, Japan) on a CFX96 Touch^TM^ Real-Time PCR Detection System (Bio-Rad, United States). DNA levels were determined using SYBR Green dye. The soybean housekeeping gene, *GmActin4* (GenBank accession no. AF049106), was used as an internal control (see Supplementary Table S1 for primer sequences). For tissue distribution analysis, the transcript levels of the *GmEF1*β gene (GenBank accession no. NM_001248778) was used as a quantitative control (see Supplementary Table S1 for primer sequences). Relative expression levels were determined using the comparative threshold method (2^-ΔΔCT^). Three biological replicates with three technical replicates of each qRT-PCR were performed.

### Subcellular Localization of GmDIR22 Protein

To determine the subcellular localization of GmDIR22, the full-length *GmDIR22* was cloned in frame at the 5′-terminus of the green fluorescent protein (GFP) coding sequence in the 35S:GFP vector using GmDIR22-GF and GmDIR22-GR (Supplementary Table S1) as the primer pair, to generate the fusion construct 35S:GmDIR22-GFP. GFP-fused GmDIR22 was transiently expressed in Arabidopsis protoplast cells, following the instructions described by Yoo et al. (2007) with minor modifications.

### Expression and Purification of GmDIR22 Fusion Protein

The full-length *GmDIR22* cDNA was framed into the *Nco*I/*Xho*I site of the pET29b(+) vector (Novagen, Germany), to generate pET29b(+)-GmDIR22. The recombinant fusion plasmid was transformed into *E. coli* BL21 (DE3) cells (TransGen Biotech, China). His-tagged GmDIR22 protein expression was induced using 0.5 mM isopropyl-β-D-thiogalactoside (IPTG) at 37°C for 6 h in LB medium containing 50 mg. mL^-1^ kanamycin. The analysis and purification of the recombinant protein were performed by SDS–PAGE.

### Analysis of Coupling Properties

Coupling assays were performed as described by Davin et al. (1997) with minor modifications. The reaction mixtures consisted of ammonium peroxydisulfate (2 μmol.mL^-1^) as an oxidant, recombinant protein (1.5 nmol.mL^-1^), and *E*-coniferyl alcohol (10 μmol.mL^-1^) in MES-NaOH buffer (0.1 M, pH 5.0) to a total volume of 250 μL. After incubation for 3 h at 30°C, the mixture was extracted three times with ethyl acetate. The extract was evaporated to dryness under vacuum, and the residue re-dissolved in 50% methanol. High Performance Liquid Chromatography (HPLC) was used to separate the substrate and product.

### Vector Construction and Soybean Transformation

The full-length coding region of *GmDIR22* was PCR amplified and inserted into the *Nco*I/*Apal*I site of pCAMBIA3301 vector containing the bar gene as the selective marker with the primer pairs *GmDIR22-oF* and *GmDIR22-oR* (Supplementary Table S1). The recombinant construct 35S:GmDIR22 was introduced into *Agrobacterium tumefaciens* strain LBA4404 using the freeze-thaw method, as described by Holsters et al. (1978). The susceptible soybean ‘Dongnong 50’ was used for gene transformation using the *Agrobacterium*-mediated method described by Paz et al. (2004). Transgenic soybean plants (T_4_) were identified by PCR amplification, and developed to T_5_ transgenic soybean plants for further analysis.

### Assays of Pathogen Responses of Transgenic Soybean Plants

The response assays of *GmDIR22*-transformed plants to *P. sojae*, the living cotyledons and detached leaves of T_5_ transgenic soybean plants were treated with pathogen inoculum following the methods described by Dou et al. (2003) with minor modifications. Non-transformed plants treated in the same way served as controls. Disease symptoms on each cotyledon were photographed after inoculation using a Nikon D7000 camera. The relative biomass of *P. sojae* in infected cotyledons was assessed after 72 h based on the transcript levels of the *P. sojae TEF1* gene (GenBank accession no. EU079791) using soybean *GmEF1*β as a reference gene, determined according to the method described by Chacón et al. (2010) (see Supplementary Table S1 for *TEF1* and *GmEF1*β primer sequences). The pathogen response assays were performed on three biological replicates with their respective three technical replicates.

### Analyses of the Lignan and Lignin Content of Transgenic Plants

Samples from air-dried stems were subjected to quantitative analysis for ligninreferred to the Klason method by Kirk and Obst (1988). The cell wall residue (% CWR) was expressed relative to lignin content. Total lignan was extracted from leaf samples referred to the method by Chen et al. (2010). The extract was dried under vacuum, and the residue was re-dissolved in ethanol buffer. The chromotropic acid spectrophotometric method as described by Lu et al. (2011) was used to determine the total lignan.

### Verification of the Antimicrobial Effects of Lignan Extracts

The antimicrobial activities of lignan extracts were assayed using the hyphal growth inhibition method, as described by Schlumbaum et al. (1986). *P. sojae* race 1 was cultured on V8 juice agar plates at 25°C for 72 h and the sterile filter-paper disks containing lignan extracts from different transgenic plants were placed around the colony. After incubation for 24 h at 25°C, the pathogen growth zones were observed. In further experiments, the inhibition of the hyphal growth of *P. sojae* with various concentrations of lignan were Researched, as described by Giannakopoulou et al. (2014) with minor modifications.

## Results

### *GmDIR22* Sequence and Bioinformatic Analysis

**FIGURE 1:**
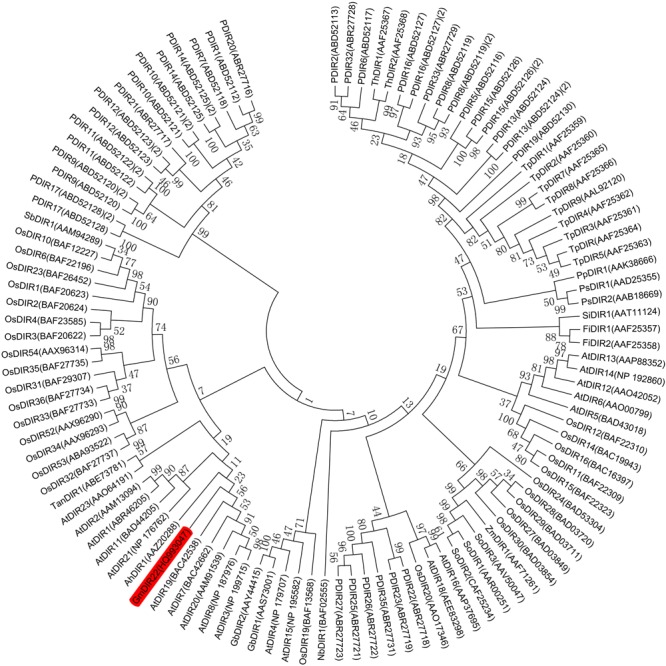
Phylogenetic tree constructed using soybean *GmDIR22* nucleotide and DIR or DIR-like nucleotide sequences from various plant species. The nucleotide sequences of 101 dirigent genes were analyzed by neighbor-joining algorithm using Mega 5.1. The stability of internal nodes was assessed by bootstrap analysis with 1000 replicates. DIR nomenclature is as follows: Ah, *Arachis hypogaea*; At, *Arabidopsis thaliana*; Fi, *Forsythia intermedia*; Gb, *Gossypium barbadense*; Gm, *Glycine max*; Nb, *Nicotiana benthamiana*; Os, *Oryza sativa*; P, *Picea glauca, Picea sitchensis*, or *P*. *glauca x engelmannii*; Pp, *Podophyllum peltatum*; Ps, *Pisum sativum*; Sb, *Sorghum bicolor*; Si, *Sesamum indicum*; So, *Saccharum officinarum*; Ta, *Tamarix androssowii*; Th, *Tsuga heterophylla*; Tp, *Thuja plicata*; and Zm, *Zea mays*.

The full-length *GmDIR22* cDNA (GenBank accession no. HQ_993047) was isolated from ‘Suinong10’ soybean by RACE. The sequence analysis showed that *GmDIR22* has an open reading frame of 567 bp and encodes a polypeptide of 188 amino acids (**Supplementary Figure S1**) with a predicted molecular mass of 20.88 kDa and a pI of 9.02. The deduced protein has a central 145 amino acid dirigent domain at amino acid residues 43–187 (**Supplementary Figure S1**). The predicted three-dimensional model of the GmDIR22 protein consists of eight β-strands, forming an eight-stranded antiparallel β-barrel (**Supplementary Figure S2**), similar to the previously reported (+)-pinoresinol-forming DIR from pea, DDR206 (Kim et al., 2015). A phylogenetic tree was constructed using the neighbor-joining algorithm based on the nucleotide sequences of *GmDIR22* that contained other members of the *DIR* family, indicating that these neighboring genes might display similar functions (**Figure 1**). A total of 101 *dir* or *dir*-like nucleotide sequences from *Glycine max* (1), *Arabidopsis thaliana* (19), *Oryza sativa* (28), *Picea glauca* (26), *Thuja plicata* (9), and an additional 18 *dir* genes identified from a variety of species, including pea, cotton, corn, and sesame, etc. (see Supplementary Table S2) were used to construct the phylogenetic tree. In the phylogenetic tree, *GmDIR22* is close to five *AtDIR* members (belonging to dir-b/d subfamily) and shares 55.15, 54.70, 53.66, 52.95, and 47.97 % nucleotide identity with *AtDIR20, AtDIR7, AtDIR19, AtDIR3*, and *AtDIR8*, respectively (**Figure 1**). We then performed sequence comparison between *GmDIR22* and the five *AtDIR* nucleotides to analyze the homology among them. The alignments of the nucleotide sequences show five well-conserved motifs (**Supplementary Figure S3**).

### Changes of *GmDIR22* Transcript Levels in Response to Stress Treatments

**FIGURE 2:**
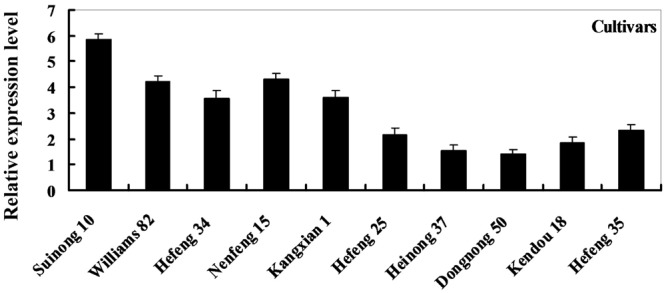
Relative expression levels of *GmDIR22* in resistant and susceptible soybean cultivars, at 48 h after *P. sojae* infection. The soybean cultivars ‘Suinong 10,’ ‘Williams 82,’ ‘Hefeng 34,’ ‘Nenfeng 15,’ and ‘Kangxian 1,’ which were resistant to *P. sojae* race 1. The soybean cultivars ‘Hefeng 25,’ ‘Heinong 37,’ ‘Dongnong 50,’ ‘Kendou 18,’ and ‘Hefeng 35’ which were susceptible to *P. sojae* race 1. Experiments were performed using three biological replicates with three technical replicates each. Bars indicated standard errors of the mean.

**FIGURE 3:**
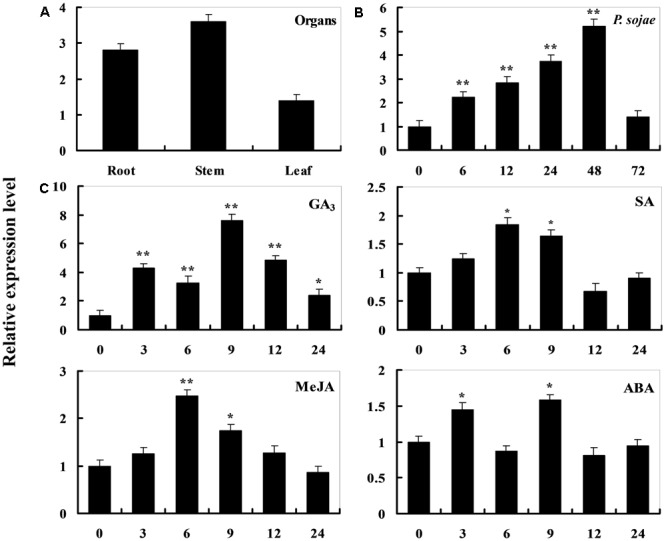
Analysis of *GmDIR22* expression patterns by qRT-PCR. **(A)** Roots, stems, and leaves were separated from 14-day-old seedlings. The amplification of the soybean *EF1* (*GmEF1*) gene was used as an internal control for data normalization. **(B)** *GmDIR22* expression in soybean leaves infected with *P. sojae.* Soybean leaves were harvested at 0, 6, 12, 24, 36, 48, and 72 h after infection. The relative *GmDIR22* transcript levels were quantified and compared between infected and mock-treated control plants at the same time point. **(C)** *GmDIR22* expression in soybean leaves in response to exogenous hormone: SA (0.5 mM), MeJA (100 μM), ABA (50 mM), and GA_3_ (50 mg.L^-1^) treatment for 0, 3, 6, 9, 12, and 24 h. The soybean *Actin* (*GmActin4*) gene was used as an internal control for data normalization. Experiments were performed using three biological replicates with three technical replicates each, and statistically analyzed using Student’s *t*-tests (^∗^*P* < 0.05, ^∗∗^*P* < 0.01). Bars indicated standard errors of the mean.

To determine the expression pattern of *GmDIR22* in different soybean cultivars, qRT-PCR was performed to examine the transcript levels of *GmDIR22* (Supplementary Table S3). The expression of *GmDIR22* was higher in resistant cultivars (‘Suinong 10,’ ‘Williams 82,’ ‘Hefeng 34,’ ‘Nenfeng 15,’ and ‘Kangxian 1’) than that in susceptible cultivars (‘Hefeng 25,’ ‘Heinong 37,’ ‘Dongnong 50,’ ‘Kendou 18,’ and ‘Hefeng 35’) (**Figure 2**). The responsiveness of *GmDIR22* mRNA transcript levels to biotic and abiotic stresses in ‘Suinong10’ soybean plants was evaluated by qRT-PCR (Supplementary Tables S5, S6, S7, S8, S9). The investigation of tissue-specific transcript abundance in ‘Suinong 10’ soybean (Supplementary Table S4) demonstrated that *GmDIR22* is constitutively and highly expressed in the stems, followed by the roots and leaves (**Figure 3A**). After *P. sojae* treatment, the levels of *GmDIR22* mRNA rapidly increases, reaching a maximum level at 48 h after treatment, followed by a decline (**Figure 3B**). *GmDIR22* expression is also induced with the treatments of GA_3_, SA, MeJA, and ABA. The levels of *GmDIR22* mRNA increases rapidly in response to GA_3_ treatment, reaching maximum levels at 9 h, followed by a rapid decline. With SA, MeJA, and ABA treatment, *GmDIR22* transcripts accumulates and reaches a maximum level at 6, 6, and 9 h, respectively, but the expression of *GmDIR22* is relatively lower than that under *P. sojae* and GA_3_ stresses (**Figure 3C**).

### Subcellular Localization of the GmDIR22 Protein

**FIGURE 4:**
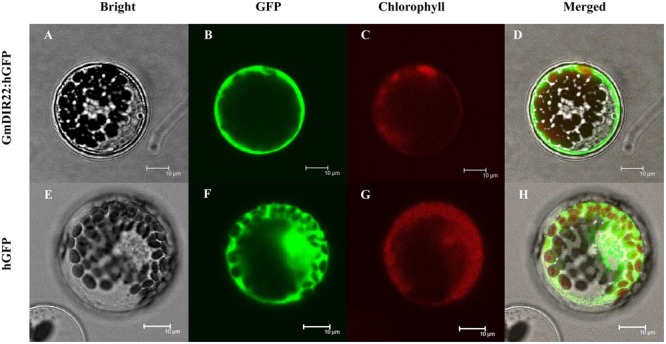
Analysis of the subcellular localization of GmDIR22-GFP protein in Arabidopsis protoplasts. GmDIR22-GFP fusion protein expression was driven by the cauliflower mosaic virus 35S promoter and transiently expressed in Arabidopsis protoplasts. Bright-field images **(A,E)**, GFP fluorescence (green) only **(B,F)**, chlorophyll autofluorescence (red) only **(C,G)**, cytoplasmic marker fluorescence localization, and combined images **(D,H)** are shown. Bars = 10 μm.

The subcellular localization of the GmDIR22 protein was analyzed by expressing a gene encoding a GmDIR22-GFP fusion protein under the control of the 35S promoter in Arabidopsis protoplasts. As shown in **Figure 4**, the observation by confocal microscopy demonstrated that GFP fluorescence is dispersed throughout the entire cells expressing the control 35S-GFP plasmid. In contrast, the GmDIR22-GFP fusion protein is localized exclusively to the membrane of the Arabidopsis cells, indicating that GmDIR22 is a membrane-localized protein.

### The Recombinant GmDIR22 Protein Directs Pinoresinol Formation

**FIGURE 5:**
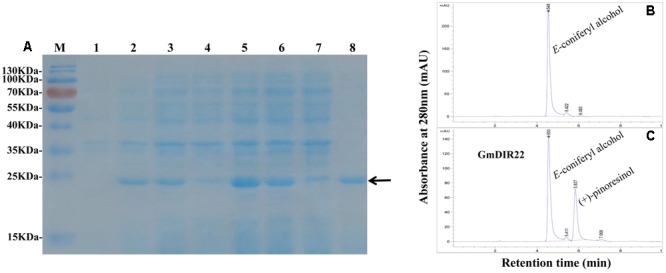
Analysis of GmDIR22 dirigent activity by HPLC. **(A)** Expression of recombinant GmDIR22 protein in *E. coli* BL21 (DE3) was induced using 0.5 mM IPTG at 37°C for 1–6 h and purified from the soluble fraction of induced cells using resin with affinity for the His-Tag. Lane 1 pET-29b(+) vector induced by IPTG for 3 h, Lane 2–7 pET29b(+)-GmDIR22 induced by IPTG for 1, 2, 3, 4, 5, and 6 h, Lane 8 purified soluble GmDIR22 protein with Nickel-CL agarose affinity chromatography. **(B)** HPLC chromatogram at 280 nm absorption of *E*-coniferyl alcohol at retention time 4.5 min. **(C)** HPLC chromatogram at 280 nm absorption of reaction mixture of *E*-coniferyl alcohol with purified soluble GmDIR22 protein, the product of (+)-pinoresinol at retention time 5.8 min.

The expression of recombinant GmDIR22 protein is remarkably enhanced by adding 0.5 mM IPTG at 37°C and increases from 1 to 6 h (**Figure 5A**). The maximum expression of the protein is achieved at 4 h. The recombinant protein was purified using His-Bind Kits (EMD Millipore, Billerica, MA, USA), and the molecular weight of the purified protein is determined as approximately 23 kDa by SDS–PAGE (**Figure 5A**). Furthermore, the dirigent ability of recombinant GmDIR22 to couple monolignols was investigated. As shown in **Figures 5B,C**, GmDIR22 could direct conversion of *E*-coniferyl alcohol into lignan (+)-pinoresinol, in the presence of ammonium peroxydisulfate as oxidant. The result is similar to that reported for FiDIR (Davin et al., 1997).

### Overexpression of *GmDIR22* Can Enhance Soybean Resistance to *P. sojae*

**FIGURE 6:**
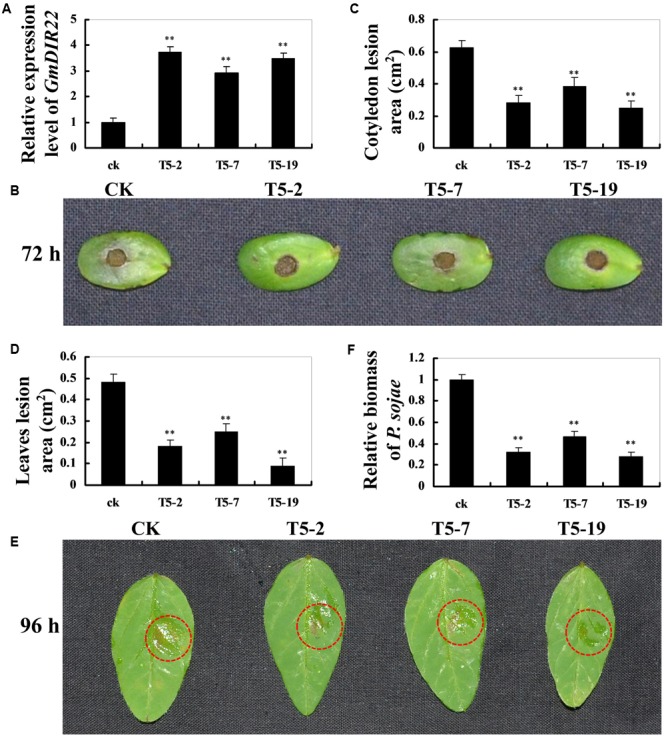
Resistance analysis of *GmDIR22* transgenic soybean plants to *P. sojae* infection. **(A)** The qRT-PCR results demonstrating the relative abundance of *GmDIR22* in transgenic soybean plants. **(B,E)** The disease symptoms on the living cotyledons and detached leaves of transgenic lines treated with *P. sojae* inoculum at 72 h and 96 h, respectively. **(C)** The relative lesion area of transgenic soybean cotyledons infected with *P. sojae* after 72 h of inoculation. **(D)** The relative lesion area of transgenic soybean leaves infection with *P. sojae* after 96 h of inoculation. **(F)** qRT-PCR analysis of *P. sojae* relative biomass based on the transcript level of the *P. sojae TEF1* gene in infected living cotyledons after 72 h of inoculation. Experiments were performed using three biological replicates with three technical replicates each, and statistically analyzed using Student’s *t*-tests (^∗^*P* < 0.05, ^∗∗^*P* < 0.01). Bars indicated standard errors of the mean.

To investigate whether overexpression of *GmDIR22* could enhance resistance to *P. sojae* in transgenic plants, the T_5_ transgenic soybean plants were selected by qRT-PCR (Supplementary Table S11) to assay the pathogen response (**Figure 6A**). The living cotyledons and detached leaves were treated with a *P. sojae* inoculum. As shown in **Figure 6B**, the cotyledons of the non-transgenic soybean plants exhibits clearer and larger water-soaked lesions compared with those of the transgenic plants after 72 h of incubation with *P. sojae*, and the lesion areas are significantly smaller in the transgenic lines than those of non-transgenic soybean plants 72 h after inoculation (*P* < 0.01) (**Figure 6C**). After 96 h incubation with *P. sojae*, the similar results are observed in the detached leaves (**Figure 6E**), and the lesion areas in transgenic soybean lines were significantly smaller than those in non-transgenic plants (*P* < 0.01) (**Figure 6D**). The biomass of *P. sojae*, based on the transcript levels of the *P. sojae TEF1* gene (Supplementary Table S10), was significantly lower in transgenic *GmDIR22*-overexpressing plants than that in non-transgenics (*P* < 0.01) (**Figure 6F**). These data indicated that the over-expression of *GmDIR22* in soybean plants in a certain extent improves their resistance to *P. sojae*.

### Overexpression of *GmDIR22* Leads to Increased Lignan Accumulation

**FIGURE 7:**
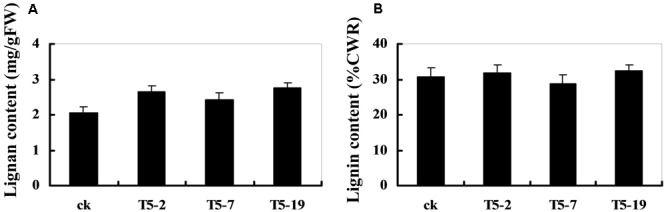
Lignan and lignin content in *GmDIR22* transgenic lines. **(A)** Lignan content. **(B)** Lignin content. Experiments were performed using three biological replicates with three technical replicates each. Bars indicated standard errors of the mean.

The dirigent phenomenon was discovered by Davin et al. (1997), and then Davin and Lewis (2000) studied the coupling of two *E*-coniferyl alcohol molecules to produce a (+)-pinoresinol as the first step in the biosynthesis of lignans. Since coniferyl and sinapyl alcohols, together with other monolignol *p*-coumaryl alcohols, are also polymerized into the macromolecule, lignin, there is some overlap in the biosynthetic pathways involved in lignans and lignin production (Davin and Lewis, 2003). Thus, the changes in the DIR expression levels may cause alterations in the lignan and lignin content of soybean plants. As shown in **Figure 7A**, the relative lignan content of transgenic plants overexpressing *GmDIR22* is significantly higher than that of non-transgenic control plants, indicating an important role for *GmDIR22* in the synthesis of lignan in soybean. In contrast, the levels of acid-insoluble lignin remain almost unchanged in transgenic plants compared with those of controls (**Figure 7B**).

### Verification of Antimicrobial Effects of Lignan Extracts

**FIGURE 8:**
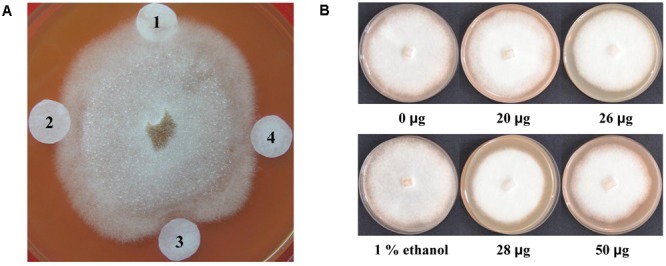
Antimicrobial activity of lignan extracts. **(A)** Inhibition of *P. sojae* race 1 growth by lignan extracts. (1) Ethanol buffer; (2) lignan extracts (20 μg) from a non-transgenic control plant; (3) lignan extracts (28 μg) from the T5-19 transgenic plant line; (4) lignan extracts (26 μg) from the T5-2 transgenic plant line. **(B)** Growth inhibition assay of *P. sojae* race 1 after 7 days of exposure of hyphal plugs to varying concentrations of lignan extracts. The experiment was performed three times.

To examine the antimicrobial effects of lignan extracts on *P. sojae* race 1, the filter-paper disks containing lignan extracts from different transgenic plants were placed around a *P. sojae* colony. After incubation for 72 h, the results showed that the lignan extracts from transgenic plant lines T5-2 and T5-19 inhibit *P. sojae* hyphal growth, leading to 1 and 2 mm inhibition zones, respectively, indicating that they exhibit an enhanced antimicrobial effect compared to those from non-transgenic plants (**Figure 8A**). To further examine the effect of lignan extracts on *P. sojae*, we conducted inhibition assays using an equal amount hyphal plugs of 1-week-old of *P. sojae* race 1, which were placed in sterilized plates with rich medium, supplemented with varying concentrations of lignans. Hyphal growth was assessed by visual inspection after 7 days, the results confirmed that the antibacterial effect gradually increased with increasing of the concentration of lignans (**Figure 8B**). These results demonstrated that lignans from soybean possess antimicrobial activity against *P. sojae*.

## Discussion

Dirigent proteins were first identified in Forsythia (Davin et al., 1997) and are considered to be responsible for an important enzymatic reaction in the production of lignan and lignin, which evolved during the adaptation of aquatic plants to terrestrial environments (Kim et al., 2002). In addition, this gene family has primary roles in defense responses, secondary metabolism, and fiber biosynthesis (Davin and Lewis, 2000; Burlat et al., 2001; Ralph et al., 2007). However, little is known about dirigent and dirigent-like proteins and their biological functions in soybean, especially the likely effect on *P. sojae* resistance in soybean. In this study, a novel dirigent gene, *GmDIR22*, that plays a positive role in soybean during infection with *P. sojae*. Here, we isolated and characterized the *GmDIR22* gene and our findings contributed to improved understanding of the function of dirigent genes in defense responses to *P. sojae* and secondary metabolism.

Dirigent proteins are members of a large family, initially subdivided into five subfamilies (DIR-a, DIR-b, DIR-c, DIR-d, and DIR-e) (Ralph et al., 2006). With the increasing numbers of DIR proteins, the DIR-b and DIR-d subfamilies are combined together with the appearance of the DIR-f and DIR-g subfamilies (Ralph et al., 2007). The phylogenetic analysis demonstrated that *GmDIR22* is close to five *AtDIR* members that had been confirmed belonging to DIR-b/d subfamily (Ralph et al., 2007), indicating that *GmDIR22* might display similar functions with DIR-b/d. Many members of which are indicated to play a role in plants responses to biotic and abiotic stresses, including H_2_O_2_, NaCl, and PEG (Guo et al., 2012), SA, GA, and MeJA (Damaj et al., 2010). SA and JA are signaling molecules associated with pathogens, insect and mechanical damage (wound) resistance. The apparent upregulation of many DIR-b/d genes in response to attacks by fungi (Williams et al., 2002; Zhu et al., 2007) and insects (Ralph et al., 2007) are of particular interest. Moreover, they have important roles in plant secondary metabolism, especially contribute to lignin formation (Zhu et al., 2007; Ma and Liu, 2015). The expression pattern determined that the transcript levels of *GmDIR22* was higher in resistant cultivars than that in susceptible cultivars with *P. sojae* infection. The result indicated that *GmDIR22* may contribute to naturally occurring resistance to the pathogen. Moreover, *GmDIR22* mRNA transcripts are strongly induced by GA_3_ stress; therefore, we speculate that *GmDIR22* may have an important role in soybean plant resistance to *P*. *sojae*, primarily through GA_3_ signaling, which is an important signal transduction component involved in activation of plant defense responses against pathogen attack (Mellersh et al., 2002). Future studies are required to determine how this process may be regulated by GA_3_ signaling.

Dirigent proteins proteins are involved in plant defenses against pathogens and are proposed to mediate the free radical coupling of monolignol plant phenols to yield the cell wall polymers, lignans and/or lignin-like compounds (Espiñeira et al., 2011; Alvarez et al., 2016). In this study, the soybean plants overexpressing the *GmDIR22* gene exhibit increased lignan accumulation, compared with controls. In contrast, the levels of acid-insoluble lignin remain almost unchanged in transgenic plants relative to those in controls. We propose two possible explanations for this result: first, GmDIR22 protein may direct the formation of lignan precursors, and is mainly, if not completely, involved in lignan biosynthesis; second, in the defense against pathogen invasion, the GmDIR22 dirigent protein may induce production of a lignin-like defense barrier, consisting of suberized or lignified metabolites associated with specialized phloem parenchyma cells or resin ducts, rather than being directly involved in the production of lignin. This is conceivable when spatial separation of lignan and lignin synthesis systems is considered, and the former occurs in the cytoplasm, while the latter takes place in the cell wall compartment (Davin et al., 2008; Bonawitz and Chapple, 2010). The subcellular localization of the GmDIR22 protein was investigated, and the results indicated that GmDIR22 is a membrane protein, suggesting that the expression of this protein in the membrane would probably be conducive when receiving stimulus signals and generate corresponding responses.

Lignans are phenylpropanoid dimers, synthesized via the phenylpropanoid pathway (Davin and Lewis, 2003). The precursor of lignan is pinoresinol, which is derived from *E*-coniferyl alcohol through the action of DIR (Burlat et al., 2001), and there is overlap between the biosynthetic pathways producing lignin and lignans (Davin and Lewis, 2003). They occur naturally in a number of plant families and are thought to have important physiological and ecological roles in interactions with insects and pathogens, due to their antifeedant activities and antibacterial (Wang et al., 1999; Harmatha and Dinan, 2003; Schroeder et al., 2006). Lignans have been studied extensively and are reported to possess a number of biological activities, including anti-inflammatory, antimicrobial, antioxidant, and anti-estrogenic properties, which may reduce the risk of cardiovascular diseases, as well as certain types of cancer (Adlercreutz and Mazur, 1997; Raffaelli et al., 2002; Arts and Hollman, 2005; Saleem et al., 2005).

In this study, lignan extracts from *GmDIR22* transgenic plants exhibit a certain effect inhibition on *P. sojae* hyphal growth. Furthermore, we determined that the recombinant GmDIR22 protein could effectively direct *E*-coniferyl alcohol coupling into (+)-pinoresinol, suggesting that overexpression of soybean *GmDIR22* is likely to promote the synthesis of lignans, leading to enhanced resistance to *P. sojae* in soybean to a certain degree. The results of analysis of lignan extracts on the inhibition of *P. sojae* hyphal growth and the living cotyledons and detached leaves of transgenic soybean plants enhancing resistance to *P. sojae* also support this hypothesis.

## Authors Contributions

Conceived and designed the experiments: PX and SZ. Performed the experiments: NL, MZ, and TL. Analyzed the data: NL, LD, QC, JW, LW, XC, CZ, and WL. Contributed reagents/materials/analysis tools: SZ and PX.

## Conflict of Interest Statement

The authors declare that the research was conducted in the absence of any commercial or financial relationships that could be construed as a potential conflict of interest.

## Footnotes

1 http://www.ncbi.nlm.nih.gov/blast

2 http://smart.embl-heidelberg.de/

3 http://www.lynnon.com/

4 http://www.megasoftware.net

5 http://www.sbg.bio.ic.ac.uk/phyre2

## Supplementary Material

FIGURE S1 Nucleotide and amino acid sequence of *GmDIR22*.

FIGURE S2 The predicted three-dimensional structure of *GmDIR22*.

FIGURE S3 Alignment of the nucleotide sequences of *GmDIR22* and the nearby 5 *AtDIR*.
