# Supplementary figures and images for: A Novel Soybean Dirigent Gene GmDIR22 Contributes to Promotion of Lignan Biosynthesis and Enhances Resistance to Phytophthora sojae
Source: Front Plant Sci. 2017 Jul 4;8:1185. doi: 10.3389/fpls.2017.01185 (PMC5495835; doi:10.3389/fpls.2017.01185)

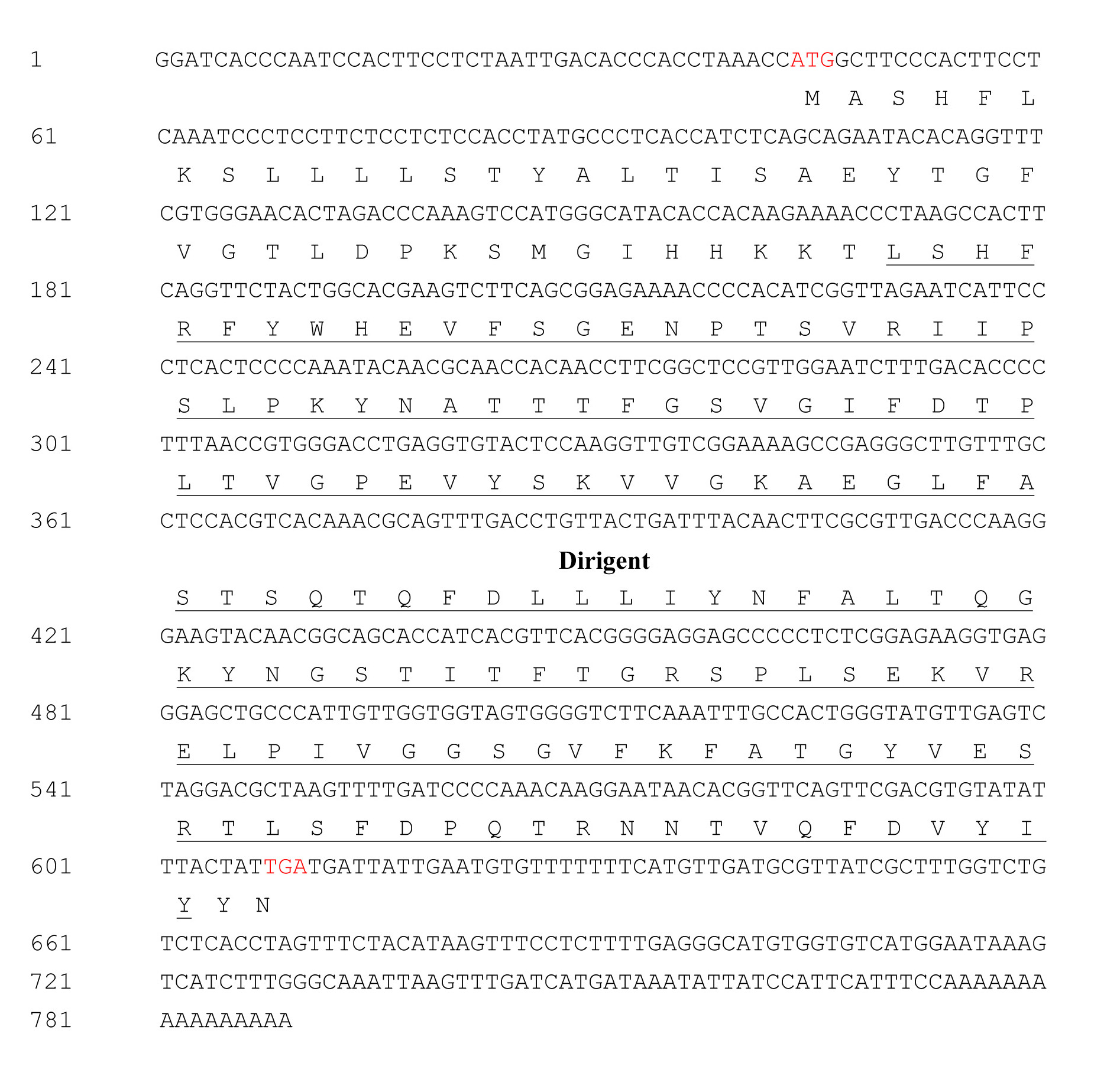

Supplement: FIGURE S1 — Nucleotide and amino acid sequence of GmDIR22. [file Image_1.JPEG]

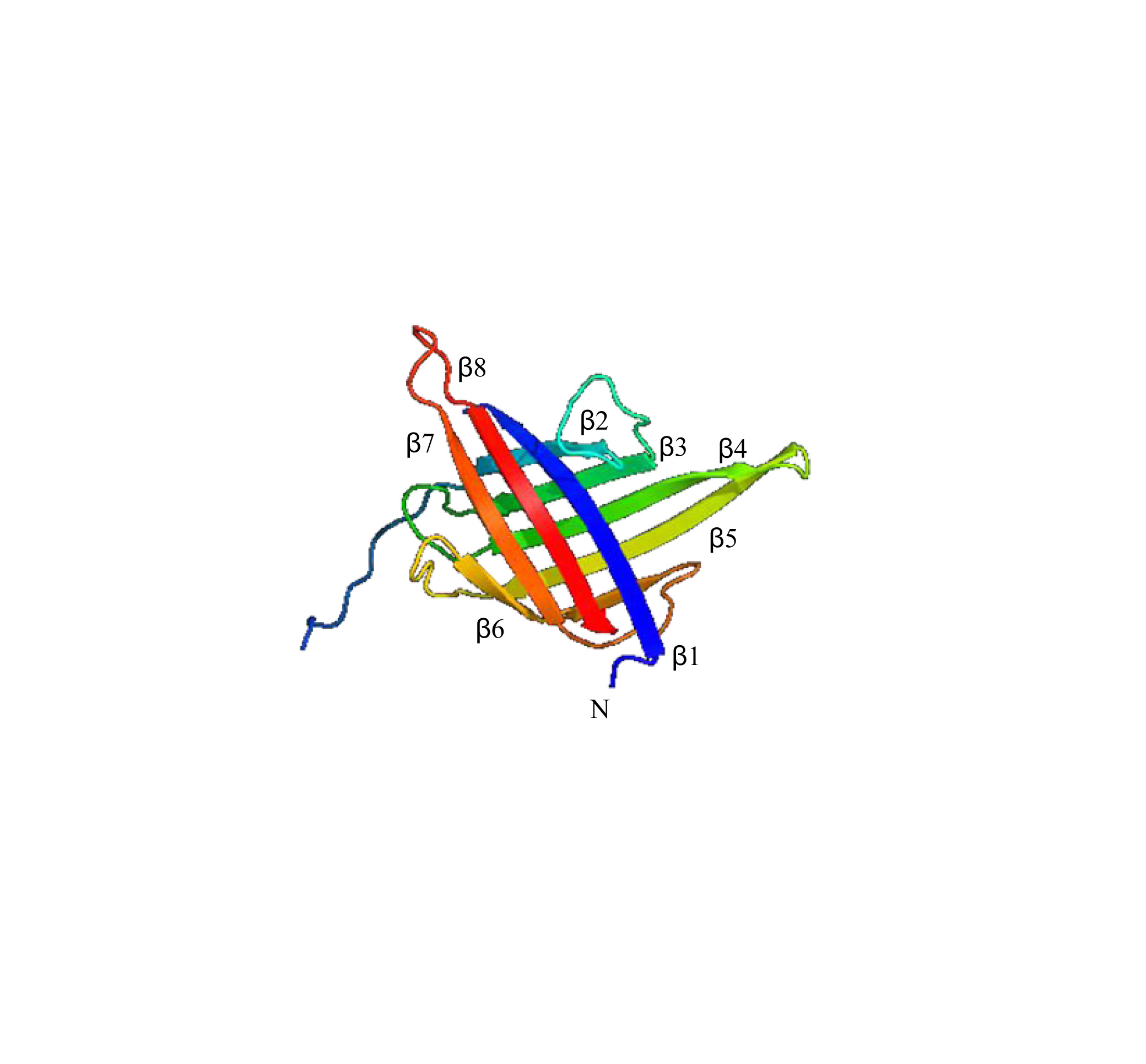

Supplement: FIGURE S2 — The predicted three-dimensional structure of GmDIR22. [file Image_2.JPEG]

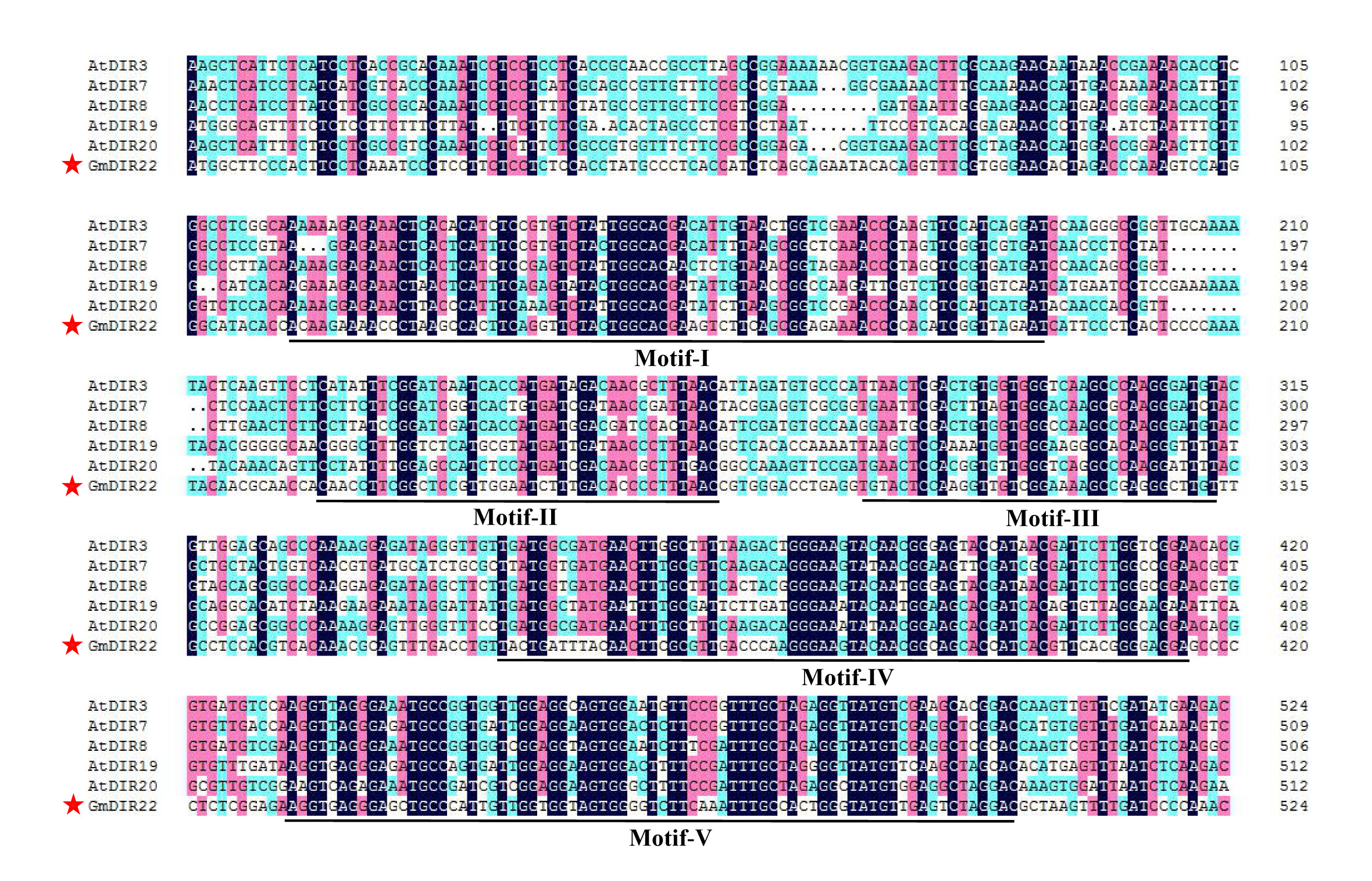

Supplement: FIGURE S3 — Alignment of the nucleotide sequences of GmDIR22 and the nearby 5 AtDIR. [file Image_3.JPEG]
