# Supplementary material for: A Novel Soybean Dirigent Gene GmDIR22 Contributes to Promotion of Lignan Biosynthesis and Enhances Resistance to Phytophthora sojae
Source: Front Plant Sci. 2017 Jul 4;8:1185. doi: 10.3389/fpls.2017.01185 (PMC5495835; doi:10.3389/fpls.2017.01185)
Supplement: Supplementary file 4 [file Table_1.DOC]

Table S1. Oligonucleotide primers used in this study.

|  | Name | Sequences |
| --- | --- | --- |
| Gene Cloning | *GSP1* | TTGTGCTTTATGCCTATCGACTTTG |
|  | *GSP2* | GGAGGGATTTGAGGAACTGGGT |
|  | *GmDIR22F* | GCTCTAGAGAGACAGACCAAAGCGATAA |
|  | *GmDIR22R* | CGAGCTCTGACACCCACCTAAACCAT |
| qPCR | *GmDIR22*-qF | GCCCATTGTTGGTGGTAGTG |
|  | *GmDIR22*-qR | CCTGAACCGTGTTATTCCTTGT |
|  | *GmActin4*F | GTGTCAGCCATACTGTCCCCATTT |
|  | *GmActin4*R | GTTTCAAGCTCTTGCTCGTAATCA |
|  | *GmEF1*β-F | CCACTGCTGAAGAAGATGATGATG |
|  | *GmEF1*β-R | AAGGACAGAAGACTTGCCACTC |
|  | *TEF1*-F | TGATCGTGCTGAACCACCC |
|  | *TEF1*-R | CGAGCGACGGTCCATCTT |
| GFP | *GmDIR22-*GF | AGATCTCATGGCTTCCCACTTCCTCAAA |
|  | *GmDIR22-*GR | ACTAGTATAGTAAATATACACGTCG |
| His | *GmDIR22*-HF | CCATGGCCATGGCTTCCCACTTCC |
|  | *GmDIR22*-HR | CTCGAGATAGTAAATATACACGTCG |
| Over-expression | *GmDIR22*-oF | CCATGGCCATGGCTTCCCACTTCC |
|  | *GmDIR22*-oR | CACGTGATAGTAAATATACACGTCG |
|  | *bar*-F | ATATCCGAGCGCCTCGTGCAT |
|  | *bar*-R | GGTCTGCACCATCGTCAACCACT |
