# Supplementary material for: A Novel Soybean Dirigent Gene GmDIR22 Contributes to Promotion of Lignan Biosynthesis and Enhances Resistance to Phytophthora sojae
Source: Front Plant Sci. 2017 Jul 4;8:1185. doi: 10.3389/fpls.2017.01185 (PMC5495835; doi:10.3389/fpls.2017.01185)
Supplement: Supplementary file 5 [file Table_2.DOC]

Table S2. The GenBank accession numbers are as follows:

AhDIR1(AAZ20288),AtDIR1(ABR46205),AtDIR2(AAM13094),AtDIR3(NP_199715),AtDIR4(NP_179707),AtDIR5(BAD43018),AtDIR6(AAO00799),AtDIR7(BAC42662),AtDIR8(NP_187976),AtDIR11(BAD44205),AtDIR12(AAO42052),AtDIR13(AAP88352),AtDIR14(NP_192860),AtDIR15(NP_195582),AtDIR16(AAP37695),AtDIR18(AEE83298),AtDIR19(BAC42538),AtDIR20(AAM91539),AtDIR21(NP_176762),AtDIR23(AAO64191),FiDIR1(AAF25357),FiDIR2(AAF25358),GbDIR1(AAS73001),GbDIR2(AAY44415),GmDIR22(HQ993047),NbDIR1(BAF02555),OsDIR1(BAF20623),OsDIR2(BAF20624),OsDIR3(BAF20622),OsDIR4(BAF23585),OsDIR6(BAF22196),OsDIR10(BAF12227),OsDIR11(BAF22309),OsDIR12(BAF22310),OsDIR14(BAC19943),OsDIR15(BAF22323),OsDIR16(BAC16397),OsDIR19(BAF13568),OsDIR20(AAO17346),OsDIR23(BAF26452),OsDIR24(BAD53304),OsDIR27(BAD03849),OsDIR28(BAD03720),OsDIR29(BAD03711),OsDIR30(BAD03854),OsDIR31(BAF29307),OsDIR32(BAF27737),OsDIR33(BAF27733),OsDIR34(AAX96293),OsDIR35(BAF27735),OsDIR36(BAF27734),OsDIR52(AAX96290),OsDIR53(ABA93522),OsDIR54(AAX96314),PDIR1(ABD52112),PDIR2(ABD52113),PDIR5(ABD52116),PDIR6(ABD52117),PDIR7(ABD52118),PDIR8(ABD52119),PDIR9(ABD52120),PDIR10(ABD52121),PDIR11(ABD52122),PDIR12(ABD52123),PDIR13(ABD52124),PDIR14(ABD52125),PDIR15(ABD52126),PDIR16(ABD52127),PDIR17(ABD52128),PDIR19(ABD52130),PDIR20(ABR27716),PDIR21(ABR27717),PDIR22(ABR27718),PDIR23(ABR27719),PDIR25(ABR27721),PDIR26(ABR27722),PDIR27(ABR27723),PDIR32(ABR27728),PDIR33(ABR27729),PDIR35(ABR27731),PpDIR1(AAK38666),PsDIR1(AAD25355),PsDIR2(AAB18669),SbDIR1(AAM94289),SiDIR1(AAT11124),SoDIR1(AAR00251),SoDIR2(CAF25234),SoDIR3(AAV50047),TanDIR1(ABE73781),ThDIR1(AAF25367),ThDIR2(AAF25368),TpDIR(AAF25364),TpDIR1(AAF25359),TpDIR2(AAF25360),TpDIR3(AAF25361),TpDIR4(AAF25362),TpDIR5(AAF25363),TpDIR7(AAF25365),TpDIR8(AAF25366),TpDIR9(AAL92120),ZmDIR1(AAF71261)
