# Supplementary material for: A Novel Soybean Dirigent Gene GmDIR22 Contributes to Promotion of Lignan Biosynthesis and Enhances Resistance to Phytophthora sojae
Source: Front Plant Sci. 2017 Jul 4;8:1185. doi: 10.3389/fpls.2017.01185 (PMC5495835; doi:10.3389/fpls.2017.01185)
Supplement: Supplementary file 6 [file Table_3.DOC]

Table S3 The raw data of relative expression levels of *GmDIR22* in resistant and susceptible soybean cultivars, at 48 h after *P. sojae* infection

| Cultivar | *EF1* | *Dir22* | Cultivar | *EF1* | *Dir22* | Cultivars | *EF1* | *Dir22* |
| --- | --- | --- | --- | --- | --- | --- | --- | --- |
| Suinong 10 | 22.39 | 19.82 | Suinong10 | 20.82 | 18.24 | Suinong 10 | 24.42 | 21.89 |
|  | 22.17 | 19.61 |  | 20.66 | 18.03 |  | 24.28 | 21.68 |
|  | 22.21 | 19.70 |  | 20.72 | 18.21 |  | 24.22 | 21.59 |
| Williams 82 | 24.08 | 21.89 | Williams 82 | 26.62 | 24.35 | Williams 82 | 25.02 | 22.84 |
|  | 23.92 | 21.89 |  | 26.82 | 24.75 |  | 24.78 | 22.70 |
|  | 23.88 | 21.89 |  | 26.34 | 24.16 |  | 24.61 | 22.63 |
| Hefeng 34 | 20.98 | 19.11 | Hefeng 34 | 22.48 | 20.78 | Hefeng 34 | 26.65 | 24.85 |
|  | 20.89 | 19.21 |  | 22.67 | 20.87 |  | 26.18 | 24.25 |
|  | 20.84 | 19.03 |  | 22.52 | 20.62 |  | 26.26 | 24.56 |
| Nenfeng 15 | 26.38 | 24.27 | Nenfeng 15 | 24.26 | 22.16 | Nenfeng 15 | 22.87 | 20.77 |
|  | 26.32 | 24.09 |  | 24.03 | 21.86 |  | 22.56 | 20.56 |
|  | 26.32 | 24.21 |  | 24.58 | 22.58 |  | 22.48 | 20.32 |
| Kangxian 1 | 24.52 | 22.59 | Kangxian 1 | 26.12 | 24.11 | Kangxian 1 | 24.12 | 22.22 |
|  | 24.46 | 22.62 |  | 25.84 | 23.93 |  | 24.57 | 22.76 |
|  | 24.33 | 22.32 |  | 26.24 | 24.43 |  | 24.59 | 22.58 |
| Hefeng 25 | 25.44 | 24.24 | Hefeng 25 | 22.98 | 21.78 | Hefeng 25 | 20.94 | 19.89 |
|  | 25.33 | 24.22 |  | 22.62 | 21.72 |  | 20.68 | 19.38 |
|  | 25.12 | 24.31 |  | 22.85 | 21.58 |  | 20.56 | 19.58 |
| Heinong 37 | 20.48 | 19.74 | Heinong 37 | 20.88 | 20.04 | Heinong 37 | 22.66 | 21.79 |
|  | 20.41 | 19.85 |  | 21.05 | 20.51 |  | 22.40 | 21.86 |
|  | 20.23 | 19.82 |  | 20.84 | 20.20 |  | 22.18 | 21.44 |
| Dongnong 50 | 23.71 | 23.48 | Dongnong 50 | 25.84 | 25.07 | Dongnong 50 | 24.24 | 23.53 |
|  | 23.58 | 23.17 |  | 25.52 | 25.01 |  | 24.63 | 24.26 |
|  | 23.94 | 23.33 |  | 25.91 | 25.46 |  | 24.68 | 24.17 |
| Kendou 18 | 22.23 | 21.33 | Kendou 18 | 24.31 | 23.23 | Kendou 18 | 20.89 | 20.19 |
|  | 22.56 | 21.56 |  | 24.18 | 23.18 |  | 21.12 | 20.22 |
|  | 22.42 | 21.72 |  | 24.43 | 23.63 |  | 21.31 | 20.31 |
| Hefeng 35 | 24.36 | 23.13 | Hefeng 35 | 22.56 | 21.43 | Hefeng 35 | 26.18 | 24.78 |
|  | 24.08 | 22.85 |  | 22.78 | 21.35 |  | 25.56 | 24.42 |
|  | 24.22 | 22.79 |  | 22.30 | 21.07 |  | 25.78 | 24.51 |
