# Supplementary material for: A Novel Soybean Dirigent Gene GmDIR22 Contributes to Promotion of Lignan Biosynthesis and Enhances Resistance to Phytophthora sojae
Source: Front Plant Sci. 2017 Jul 4;8:1185. doi: 10.3389/fpls.2017.01185 (PMC5495835; doi:10.3389/fpls.2017.01185)
Supplement: Supplementary file 7 [file Table_4.DOC]

Table S4 The raw data of relative expression level of *GmDir22* in ‘Suinong 10’ soybean organs

| Organ | *EF1* | *Dir22* | Organ | *EF1* | *Dir22* | Organ | *EF1* | *Dir22* |
| --- | --- | --- | --- | --- | --- | --- | --- | --- |
| Root | 23.74 | 22.15 | Root | 22.47 | 20.98 | Root | 20.32 | 18.86 |
|  | 23.51 | 22.12 |  | 22.37 | 20.78 |  | 20.57 | 18.92 |
|  | 23.58 | 22.09 |  | 22.64 | 21.25 |  | 20.61 | 19.12 |
| Stem | 20.78 | 18.93 | Stem | 23.2 | 21.45 | Stem | 22.62 | 20.77 |
|  | 21.16 | 19.41 |  | 23.61 | 21.66 |  | 22.48 | 20.53 |
|  | 21.22 | 19.27 |  | 23.47 | 21.63 |  | 22.47 | 20.72 |
| Leaf | 24.52 | 23.93 | Leaf | 21.38 | 20.99 | Leaf | 22.13 | 21.82 |
|  | 24.54 | 24.05 |  | 21.47 | 20.88 |  | 22.18 | 21.55 |
|  | 24.11 | 23.87 |  | 21.59 | 21.40 |  | 22.65 | 22.06 |
