# Supplementary material for: A Novel Soybean Dirigent Gene GmDIR22 Contributes to Promotion of Lignan Biosynthesis and Enhances Resistance to Phytophthora sojae
Source: Front Plant Sci. 2017 Jul 4;8:1185. doi: 10.3389/fpls.2017.01185 (PMC5495835; doi:10.3389/fpls.2017.01185)
Supplement: Supplementary file 8 [file Table_5.DOC]

Table S5 The raw data of relative expression level of *GmDir22* in leaves of ‘Suinong 10’ soybean with GA_3_ treatment

| Time | *Actin* | *Dir22* | Time | *Actin* | *Dir22* | Time | *Actin* | *Dir22* |
| --- | --- | --- | --- | --- | --- | --- | --- | --- |
| 0 h | 20.42 | 19.94 | 0 h | 21.38 | 20.41 | 0 h | 20.87 | 20.39 |
|  | 20.59 | 19.61 |  | 21.56 | 21.12 |  | 20.66 | 20.21 |
|  | 20.61 | 20.13 |  | 21.68 | 21.02 |  | 20.94 | 20.01 |
| 3 h | 21.82 | 19.25 | 3 h | 22.61 | 19.51 | 3 h | 22.32 | 19.7 |
|  | 21.97 | 19.11 |  | 22.12 | 19.76 |  | 22.54 | 20.02 |
|  | 21.65 | 19.13 |  | 22.67 | 19.95 |  | 22.58 | 19.72 |
| 6 h | 23.02 | 20.70 | 6 h | 23.18 | 20.56 | 6 h | 24.12 | 21.71 |
|  | 23.18 | 20.46 |  | 22.94 | 20.55 |  | 23.89 | 21.57 |
|  | 22.92 | 20.42 |  | 22.95 | 20.49 |  | 23.78 | 21.22 |
| 9 h | 24.71 | 21.37 | 9 h | 24.76 | 21.05 | 9 h | 24.62 | 21.18 |
|  | 24.85 | 21.18 |  | 24.38 | 21.11 |  | 24.48 | 21.21 |
|  | 24.42 | 21.11 |  | 24.82 | 21.25 |  | 24.58 | 20.81 |
| 12 h | 23.38 | 20.64 | 12 h | 23.65 | 20.61 | 12 h | 23.12 | 20.58 |
|  | 23.12 | 20.08 |  | 23.33 | 20.64 |  | 23.05 | 20.36 |
|  | 23.21 | 20.57 |  | 23.62 | 20.78 |  | 23.71 | 20.67 |
| 24 h | 21.14 | 19.35 | 24 h | 22.48 | 20.56 | 24 h | 21.78 | 19.99 |
|  | 21.18 | 19.25 |  | 22.28 | 20.53 |  | 21.58 | 19.73 |
|  | 21.32 | 19.63 |  | 22.61 | 20.72 |  | 21.64 | 19.65 |
