# Supplementary material for: A Novel Soybean Dirigent Gene GmDIR22 Contributes to Promotion of Lignan Biosynthesis and Enhances Resistance to Phytophthora sojae
Source: Front Plant Sci. 2017 Jul 4;8:1185. doi: 10.3389/fpls.2017.01185 (PMC5495835; doi:10.3389/fpls.2017.01185)
Supplement: Supplementary file 9 [file Table_6.DOC]

Table S6 The raw data of relative expression level of *GmDir22* in leaves of ‘Suinong 10’ soybean with SA treatment

| Time | *Actin* | *Dir22* | Time | *Actin* | *Dir22* | Time | *Actin* | *Dir22* |
| --- | --- | --- | --- | --- | --- | --- | --- | --- |
| 0 h | 19.76 | 19.25 | 0 h | 20.48 | 19.90 | 0 h | 20.12 | 19.64 |
|  | 19.91 | 19.23 |  | 20.56 | 19.88 |  | 20.23 | 19.75 |
|  | 19.88 | 19.31 |  | 20.42 | 19.94 |  | 20.37 | 19.73 |
| 3 h | 20.32 | 19.51 | 3 h | 21.22 | 20.41 | 3 h | 22.35 | 21.54 |
|  | 20.47 | 19.56 |  | 21.35 | 20.44 |  | 22.46 | 21.52 |
|  | 20.62 | 19.61 |  | 21.51 | 20.62 |  | 22.48 | 21.57 |
| 6 h | 22.45 | 21.01 | 6 h | 22.92 | 21.45 | 6 h | 24.24 | 22.89 |
|  | 22.39 | 20.99 |  | 22.86 | 21.28 |  | 24.36 | 22.91 |
|  | 22.61 | 21.16 |  | 22.83 | 21.28 |  | 24.55 | 23.13 |
| 9 h | 24.12 | 22.89 | 9 h | 23.22 | 21.79 | 9 h | 23.08 | 21.85 |
|  | 24.15 | 22.82 |  | 23.18 | 21.75 |  | 23.12 | 21.79 |
|  | 24.32 | 22.89 |  | 23.18 | 22.06 |  | 23.33 | 21.99 |
| 12 h | 20.20 | 19.95 | 12 h | 22.32 | 22.30 | 12 h | 22.68 | 22.46 |
|  | 20.12 | 20.05 |  | 22.47 | 22.45 |  | 22.65 | 22.42 |
|  | 20.43 | 20.32 |  | 22.68 | 22.36 |  | 22.48 | 22.49 |
| 24 h | 21.56 | 20.98 | 24 h | 21.73 | 21.20 | 24 h | 21.56 | 21.43 |
|  | 21.38 | 20.92 |  | 21.56 | 21.13 |  | 21.57 | 21.14 |
|  | 21.22 | 20.81 |  | 21.68 | 21.25 |  | 21.69 | 21.16 |
