# Supplementary material for: A Novel Soybean Dirigent Gene GmDIR22 Contributes to Promotion of Lignan Biosynthesis and Enhances Resistance to Phytophthora sojae
Source: Front Plant Sci. 2017 Jul 4;8:1185. doi: 10.3389/fpls.2017.01185 (PMC5495835; doi:10.3389/fpls.2017.01185)
Supplement: Supplementary file 10 [file Table_7.DOC]

Table S7 The raw data of relative expression level of *GmDir22* in leaves of ‘Suinong 10’ soybean with ABA treatment

| Time | *Actin* | *Dir22* | Time | *Actin* | *Dir22* | Time | *Actin* | *Dir22* |
| --- | --- | --- | --- | --- | --- | --- | --- | --- |
| 0 h | 20.38 | 19.80 | 0 h | 19.78 | 19.3 | 0 h | 20.62 | 20.04 |
|  | 20.22 | 19.74 |  | 19.76 | 19.18 |  | 20.41 | 19.93 |
|  | 20.47 | 19.79 |  | 19.82 | 19.14 |  | 20.66 | 19.98 |
| 3 h | 23.42 | 22.41 | 3 h | 22.38 | 21.21 | 3 h | 23.02 | 22.01 |
|  | 23.55 | 22.44 |  | 22.45 | 21.34 |  | 22.86 | 21.76 |
|  | 23.58 | 22.37 |  | 22.58 | 21.23 |  | 22.96 | 21.85 |
| 6 h | 21.65 | 21.49 | 6 h | 20.73 | 20.72 | 6 h | 21.22 | 20.86 |
|  | 21.78 | 21.42 |  | 20.77 | 20.41 |  | 20.95 | 20.69 |
|  | 21.68 | 21.37 |  | 20.74 | 20.48 |  | 20.89 | 20.71 |
| 9 h | 24.32 | 22.96 | 9 h | 23.65 | 22.58 | 9 h | 24.28 | 23.07 |
|  | 24.33 | 23.07 |  | 23.76 | 22.5 |  | 24.48 | 23.28 |
|  | 24.36 | 23.10 |  | 23.90 | 22.41 |  | 24.72 | 23.31 |
| 12 h | 22.22 | 21.89 | 12 h | 21.78 | 21.45 | 12 h | 22.28 | 22.05 |
|  | 22.34 | 22.11 |  | 21.76 | 21.53 |  | 22.52 | 22.29 |
|  | 22.33 | 22.20 |  | 21.72 | 21.59 |  | 22.17 | 22.24 |
| 24 h | 21.64 | 20.92 | 24 h | 20.64 | 19.98 | 24 h | 21.11 | 20.59 |
|  | 21.68 | 21.08 |  | 20.87 | 20.25 |  | 21.32 | 20.70 |
|  | 21.58 | 21.01 |  | 20.85 | 20.33 |  | 21.42 | 20.75 |
