# Supplementary material for: A Novel Soybean Dirigent Gene GmDIR22 Contributes to Promotion of Lignan Biosynthesis and Enhances Resistance to Phytophthora sojae
Source: Front Plant Sci. 2017 Jul 4;8:1185. doi: 10.3389/fpls.2017.01185 (PMC5495835; doi:10.3389/fpls.2017.01185)
Supplement: Supplementary file 11 [file Table_8.DOC]

Table S8 The raw data of relative expression level of *GmDir22* in leaves of ‘Suinong 10’ soybean with MeJA treatment

| Time | *Actin* | *Dir22* | Time | *Actin* | *Dir22* | Time | *Actin* | *Dir22* |
| --- | --- | --- | --- | --- | --- | --- | --- | --- |
| 0 h | 19.85 | 19.17 | 0 h | 19.64 | 19.02 | 0 h | 20.42 | 19.74 |
|  | 19.86 | 19.28 |  | 19.87 | 19.39 |  | 20.35 | 19.87 |
|  | 19.78 | 19.30 |  | 19.86 | 19.12 |  | 20.52 | 19.84 |
| 3 h | 20.45 | 19.56 | 3 h | 21.22 | 20.42 | 3 h | 21.55 | 20.75 |
|  | 20.62 | 19.72 |  | 21.54 | 20.64 |  | 21.41 | 20.45 |
|  | 20.78 | 19.81 |  | 21.58 | 20.58 |  | 21.49 | 20.57 |
| 6 h | 23.24 | 21.25 | 6 h | 24.45 | 22.46 | 6 h | 23.89 | 22.01 |
|  | 23.22 | 21.33 |  | 24.43 | 22.64 |  | 24.08 | 22.29 |
|  | 23.46 | 21.56 |  | 24.66 | 22.69 |  | 24.21 | 22.22 |
| 9 h | 22.63 | 21.24 | 9 h | 21.88 | 20.49 | 9 h | 22.59 | 21.21 |
|  | 22.48 | 21.19 |  | 21.78 | 20.36 |  | 22.57 | 21.28 |
|  | 22.49 | 21.10 |  | 21.92 | 20.42 |  | 22.47 | 20.88 |
| 12 h | 21.65 | 20.62 | 12 h | 22.28 | 21.35 | 12 h | 20.58 | 19.55 |
|  | 21.5 | 20.67 |  | 22.31 | 21.32 |  | 20.79 | 19.81 |
|  | 21.85 | 20.79 |  | 22.37 | 21.44 |  | 20.48 | 19.65 |
| 24 h | 20.74 | 20.29 | 24 h | 20.45 | 20.30 | 24 h | 19.64 | 19.29 |
|  | 20.65 | 20.40 |  | 20.26 | 20.01 |  | 19.47 | 19.02 |
|  | 20.92 | 20.45 |  | 20.4 | 19.85 |  | 19.78 | 19.23 |
