# Supplementary material for: A Novel Soybean Dirigent Gene GmDIR22 Contributes to Promotion of Lignan Biosynthesis and Enhances Resistance to Phytophthora sojae
Source: Front Plant Sci. 2017 Jul 4;8:1185. doi: 10.3389/fpls.2017.01185 (PMC5495835; doi:10.3389/fpls.2017.01185)
Supplement: Supplementary file 12 [file Table_9.DOC]

Table S9 The raw data of relative expression level of *GmDir22* in leaves of ‘Suinong 10’ soybean with *P. sojae* treatment

| Time | *Actin* | *Dir22* | Time | *Actin* | *Dir22* | Time | *Actin* | *Dir22* |
| --- | --- | --- | --- | --- | --- | --- | --- | --- |
| 0 h | 22.61 | 22.12 | 0 h | 23.45 | 22.67 | 0 h | 21.85 | 21.11 |
|  | 22.12 | 21.28 |  | 23.68 | 23.10 |  | 21.98 | 21.41 |
|  | 22.12 | 21.53 |  | 23.62 | 23.34 |  | 21.68 | 21.4 |
| 6 h | 23.29 | 21.52 | 6 h | 22.92 | 20.90 | 6 h | 22.33 | 20.51 |
|  | 23.27 | 21.49 |  | 22.88 | 21.26 |  | 22.49 | 20.85 |
|  | 23.23 | 21.56 |  | 22.83 | 21.31 |  | 22.56 | 20.94 |
| 12 h | 23.49 | 21.27 | 12 h | 24.18 | 22.06 | 12 h | 22.98 | 20.76 |
|  | 23.68 | 21.30 |  | 24.33 | 22.11 |  | 23.32 | 21.2 |
|  | 23.16 | 21.04 |  | 24.26 | 22.34 |  | 23.05 | 21.06 |
| 24 h | 24.32 | 21.82 | 24 h | 23.87 | 21.27 | 24 h | 24.78 | 22.23 |
|  | 24.24 | 21.64 |  | 23.95 | 21.41 |  | 24.63 | 22.13 |
|  | 24.01 | 21.61 |  | 24.12 | 21.82 |  | 24.68 | 22.42 |
| 48 h | 22.65 | 19.69 | 48 h | 25.72 | 22.51 | 48 h | 25.12 | 22.09 |
|  | 22.85 | 19.69 |  | 25.48 | 22.37 |  | 24.87 | 21.91 |
|  | 22.27 | 19.31 |  | 25.28 | 22.71 |  | 24.86 | 22.12 |
| 72 h | 21.01 | 19.86 | 72 h | 21.18 | 20.23 | 72 h | 21.66 | 20.61 |
|  | 20.56 | 19.71 |  | 21.2 | 20.05 |  | 21.82 | 20.87 |
|  | 20.36 | 19.41 |  | 21.46 | 20.49 |  | 21.61 | 20.56 |
