# Supplementary material for: A Novel Soybean Dirigent Gene GmDIR22 Contributes to Promotion of Lignan Biosynthesis and Enhances Resistance to Phytophthora sojae
Source: Front Plant Sci. 2017 Jul 4;8:1185. doi: 10.3389/fpls.2017.01185 (PMC5495835; doi:10.3389/fpls.2017.01185)
Supplement: Supplementary file 13 [file Table_10.DOC]

Table S10 The raw data of relative expression level of *GmDIR22* in transgenic soybean plants

| Plants | *EF1* | *Dir22* | Plants | *EF1* | *Dir22* | Plants | *EF1* | *Dir22* |
| --- | --- | --- | --- | --- | --- | --- | --- | --- |
| CK | 22.32 | 21.99 | CK | 21.88 | 21.42 | CK | 20.45 | 20.09 |
|  | 22.66 | 22.10 |  | 21.76 | 21.50 |  | 20.57 | 20.11 |
|  | 22.48 | 22.22 |  | 21.85 | 21.46 |  | 20.62 | 20.36 |
| T5-2 | 23.22 | 20.86 | T5-2 | 22.55 | 20.17 | T5-2 | 22.58 | 20.20 |
|  | 23.56 | 21.02 |  | 22.59 | 20.31 |  | 22.71 | 20.31 |
|  | 23.35 | 21.17 |  | 22.62 | 20.31 |  | 22.32 | 20.15 |
| T5-7 | 23.32 | 21.40 | T5-7 | 23.72 | 21.61 | T5-7 | 22.89 | 20.87 |
|  | 23.48 | 21.52 |  | 23.51 | 21.76 |  | 22.89 | 20.92 |
|  | 23.34 | 21.52 |  | 23.70 | 21.68 |  | 22.77 | 20.85 |
| T5-19 | 24.22 | 22.16 | T5-19 | 25.46 | 23.20 | T5-19 | 24.22 | 22.11 |
|  | 24.44 | 22.08 |  | 25.22 | 23.24 |  | 24.45 | 22.15 |
|  | 24.38 | 22.28 |  | 25.41 | 23.25 |  | 24.01 | 21.96 |
