# Supplementary material for: A Novel Soybean Dirigent Gene GmDIR22 Contributes to Promotion of Lignan Biosynthesis and Enhances Resistance to Phytophthora sojae
Source: Front Plant Sci. 2017 Jul 4;8:1185. doi: 10.3389/fpls.2017.01185 (PMC5495835; doi:10.3389/fpls.2017.01185)
Supplement: Supplementary file 14 [file Table_11.DOC]

Table S11 The raw data of qRT-PCR analysis of *P. sojae* relative biomass based on the transcript level of the *P. sojae* *TEF1* gene in infected living cotyledons after 72 h of inoculation

| Plants | *EF1* | *TEF1* | Plants | *EF1* | *TEF1* | Plants | *EF1* | *TEF1* |
| --- | --- | --- | --- | --- | --- | --- | --- | --- |
| CK | 21.41 | 25.63 | CK | 20.56 | 24.56 | CK | 22.45 | 26.75 |
|  | 21.62 | 25.86 |  | 20.36 | 24.66 |  | 22.66 | 26.86 |
|  | 21.35 | 25.59 |  | 20.22 | 24.48 |  | 22.63 | 27.03 |
| T5-2 | 22.45 | 25.05 | T5-2 | 21.86 | 24.36 | T5-2 | 24.54 | 27.34 |
|  | 22.68 | 25.21 |  | 21.36 | 24.53 |  | 24.6 | 27.65 |
|  | 22.42 | 25.41 |  | 21.82 | 24.62 |  | 24.72 | 27.42 |
| T5-7 | 24.21 | 27.45 | T5-7 | 22.83 | 25.57 | T5-7 | 25.18 | 28.42 |
|  | 24.94 | 27.68 |  | 22.71 | 25.79 |  | 25.22 | 28.36 |
|  | 24.96 | 27.70 |  | 22.33 | 25.57 |  | 25.62 | 28.51 |
| T5-19 | 20.88 | 23.27 | T5-19 | 23.92 | 25.61 | T5-19 | 24.86 | 27.05 |
|  | 20.92 | 23.11 |  | 23.22 | 25.91 |  | 24.78 | 27.41 |
|  | 20.56 | 23.55 |  | 23.57 | 25.96 |  | 24.65 | 27.24 |
